# Preparation and properties of hydrochars from macadamia nut shell via hydrothermal carbonization

**DOI:** 10.1098/rsos.181126

**Published:** 2018-10-03

**Authors:** Fangyu Fan, Zongling Yang, Han Li, Zhengjun Shi, Huan Kan

**Affiliations:** Key Laboratory for Forest Resources Conservation and Utilisation in the Southwest Mountains of China, Ministry of Education, and School of Light Industry and Food Engineering, Southwest Forestry University, Kunming, Yunnan 650224, People's Republic of China

**Keywords:** macadamia nut shell, hydrothermal carbonization, hydrochar, property

## Abstract

Macadamia nut shell (MNS) is a type of waste lignocellulose obtained from macadamia nut production processing. Large MNS wastes caused serious resource waste and environmental pollution. So, preparation of hydrochars from MNS via hydrothermal carbonization (HTC) is of great significance. HTC of MNS was conducted to study the effect of process parameters, including HTC temperature (180–260°C) and residence time (60–180 min) on the properties of hydrochars. Results showed that the increase in HTC temperature and residence time decreased the mass yield of hydrochars and increased the high heating value of hydrochars. Furthermore, the C content of hydrochars increased, whereas the H and O contents decreased. Mass yield of hydrochar is 46.59%, energy yield is 64.55% and the higher heating value is 26.02 MJ kg^−1^ at a temperature of 260°C and time of 120 min. The surface structure of hydrochars was rougher compared with that of MNS as observed via scanning electron microscopy. The chemical and combustion behaviour of MNS and hydrochars was analysed by Fourier transform infrared spectroscopy, and thermogravimetric analysis indicated that decarboxylation and dehydration reactions were the predominant pathways during the HTC process. Results showed that HTC can facilitate the transformation of MNS into solid fuel.

## 1. Introduction

Energy demand continues to increase with the growth of the world population [1,2]. Meanwhile, the demand for energy in industrialization and urbanization leads to severe energy shortage and environmental pollution [3–5]. Thus, biomass energy was developed to solve these problems because biomass is an eco-friendly, renewable feedstock for producing various bio-based products that can be used to replace fossil fuels for consumption [6,7].

Macadamia nut shell (MNS) is a type of waste lignocellulose obtained from macadamia nut production processing. Approximately 44 000 metric tons (kernel) is produced worldwide [8]. Thus, the comprehensive utilization of MNS is an important issue to be solved in the development of the macadamia nut industry. At present, discarding and burning, which have caused serious resource waste and environmental pollution, are the main routes to deal with MNS. Therefore, conversion of MNS into a resource with increased value is an important pressing concern. The use of MNS has been investigated and explored in various approaches. Cholake *et al*. [9] produced a wood–plastic composite using waste MNS and automotive plastic. Zheng *et al*. [10] explored a new method to produce a high-performance sodium-ion battery enhanced by MNS-derived hard carbon anode. Rodrigues *et al*. [11] produced activated carbon-derived MNS for potential use as an adsorbent for phenol removal. Li *et al*. [12] investigated nitrogen-doped activated carbon from MNS and its application in supercapacitors. However, studies on the energy utilization of MNS are rarely reported. In these respects, a related study has been conducted [13,14]. The results show that prepared biochar from MNS offers an important application prospect.

Hydrothermal carbonization (HTC) is one of the technologies for biomass energy utilization [15]. Compared with other techniques, such as pyrolysis and torrefaction [15–17], HTC exhibits distinct advantages, such as high conversion efficiency, simplicity and relatively mild reaction conditions; in addition, the approach eliminates energy-intensive drying content [18–21]. Nizamuddin *et al*. [18] investigated the chemical, dielectric and structural characteristics of optimized hydrochar produced from the HTC of palm shell and analysed the effects of conditions on the properties of hydrochar. Lei *et al*. [19] produced hydrochar from corncob residues, and the results indicated that the HTC method is a promising approach to upgrade corncob residues to a high heating value hydrochar. Wang *et al*. [20] researched the effect of pH value of woody biomass at 200 and 250°C from HTC. During HTC, feedstock can be converted into a coal-like product called hydrochar. Yang *et al*. [22] produced hydrochar from bamboo using HTC. The results indicated that HTC can increase the fuel properties and combustion behaviour of bamboo. Gao *et al*. [23] used eucalyptus bark as a feedstock for producing hydrochar via HTC by varying temperature within the range of 220–300°C and varying residence time from 2 to 10 h. In addition, various substances, such as poly [24], corn stalk [25], municipal solid waste [26], fish waste [27] and pine [28], have been used as feedstocks to obtain solid fuel. The results show that the properties of hydrochar, such as yield, fixed carbon (FC) content, and surface structure, were altered under different conditions. The preparation and properties of hydrochars from MNS via HTC can provide a reference for the comprehensive utilization of macadamia nut waste.

HTC was used to convert MNS into high-quality energy resources on the basis of the previously discussed successful research. To the best of our knowledge, no studies regarding the preparation and characterization of hydrochar from MNS via HTC have been conducted. This study aims to analyse the effects of HTC reaction temperature and residence time on the chemical, structural and combustion properties of hydrochar from MNS in terms of proximate analysis, ultimate analysis, mass yield, energy yield, higher heating value (HHV), scanning electron microscopy (SEM), Fourier transform infrared spectroscopy (FTIR) and thermogravimetric analysis (TGA).

## 2. Material and methods

### 2.1. Materials

MNSs were collected from Yunnan, China. The shells were washed with deionized water three times and stored in an oven at 105°C for 24 h to dry. The dried MNSs were milled to a size of under 100 meshes (0.16 mm) as test samples, and stored in a sealed bag.

### 2.2. Hydrothermal carbonization experiments

HTC experiments were performed in a laboratory-scale, semi-batch, 500 ml Parr autoclave reactor (Moline, IL, USA), equipped with a temperature controller and indicator module of pressure and stirrer rate. In each batch experiment, approximately 50 g of MNS powder was loaded with 400 ml of deionized water into the reactor at room temperature. The reactor was sealed, and flooded with nitrogen under high pressure three times to discharge oxygen from the reactor. Subsequently, the reactor was heated to the desired temperature (180, 220 and 260°C) and maintained at 120 min. Furthermore, the varied residence times of 60, 120 and 180 min were investigated at 220°C. The heating rate was set at 8°C min^−1^, and the stirring rate was 100 r.p.m. After the reaction was completed, the reactor was immediately immersed in water for cooling to room temperature. The hydrochar was filtered via a glass filter, oven-dried at 105°C for 24 h and then stored in a sealed bag. The hydrochar from MNS were designated as MNSXX–YY, where XX and YY represented temperature and residence time, respectively.

### 2.3. Research methodology

Ultimate analysis (C, H, N and S) was conducted using a Vario EL III elemental analyzer (Elementar, Germany), and oxygen content was calculated by difference based on the following equation.

2.1 O(% ) =100(% ) −C(% ) −H(% ) −N(% ) −S(% ) −ash(% ) 

The HHVs of samples were estimated using a Parr 6300 bomb calorimeter (USA). Proximate analysis was conducted using a 5E-MAG6600 automatic proximate analyzer (China). Energy densification, mass yield and energy yield were calculated using equations (2.2)–(2.4), respectively [15,19].

2.2 $$\text{energy densification = }\frac{\text{HH}{\text{V}}_{\mathrm{hydrochar}}}{\text{HH}{\text{V}}_{\mathrm{MNS}}},$$

2.3 $$\text{mass yield (\%) = }\left(\frac{m_{\mathrm{hydrochar}}}{m_{\mathrm{MNS}}}\right)\times 100$$

2.4 $$\;\text{and}\;\text{energy yield (\%) = }\left(\frac{\text{HH}{\text{V}}_{\mathrm{hydrochar}}}{\text{HH}{\text{V}}_{\mathrm{MNS}}}\right)\times \text{mass yield}.$$

The functional groups were determined by FTIR spectra (Magna-IR 560 ESP Thermo Nicolet Waltham, MA, USA). The spectrum recorded a wavenumber that ranges from 400 to 4000 cm^−1^. Samples discs were prepared by mixing the dried sample powder and KBr powder at a sample/KBr ratio of 1 : 200. For SEM measurements, the samples were sputter-coated with Pt and examined with a JSM-6490LV scanning electron microscope (JEOL, Japan). The Brunauer-Emmett-Teller (BET) surface of hydrochar from MNS was analysed using BET (Micrometrics ASAP 2020, Norcross, USA).

The combustion behaviour of the samples was evaluated by TGA (Netzsch, Germany) within a temperature range of ambient temperature to 900°C, at a rate of 20°C min^−1^. The gas flow rate was maintained at 60 ml min^−1^ (N_2_/O_2_ = 4 : 1).

## 3. Results and discussion

### 3.1. Effect of process parameters

Energy densification, mass yield and energy yield of hydrochars from different conditions were summarized in table 1. The results indicated that HTC temperature markedly effected the mass yield of hydrochar. With the increase in HTC temperature from 180 to 260°C, the mass yield decreased significantly from 72.42% (MNS180–120) to 46.59% (MNS260–120) possibly due to the hydrolysis and decomposition of hemicellulose, cellulose and a portion of lignin [29,30]. During HTC, the pyrolysis temperature of cellulose, hemicellulose and lignin is lower than the experimental temperature, but cellulose was sharply hydrolysed beyond 200°C, hemicellulose was denatured at approximately 180°C and the main stages of lignin degraded at above 300°C [29,31]. Moreover, heated subcritical water promoted the dissolution of the organic compounds of the samples with the increase in reaction temperature, thereby resulting in the decrease in mass yield [23]. Several researchers believed that the dielectric constant of solution decreases with the increase in carbonization temperature, including water to behave similarly to a polar organic solvent. Therefore, high HTC temperature promoted the dissolution of small organic compounds from the samples, resulting in the decrease in the mass yield of hydrochar [32,33]. A similar trend was observed for the solid hydrochar produced from potato waste at 180–300°C for 1 h [32], and corncob residues at 190–370°C for 1.5 h [19]. Compared with that of HTC temperature, the effect of different residence times on the mass yield of hydrochar was less notable because the slow heating rate lengthened the time to achieve the desired temperature. Therefore, an equilibrium state among decarbonization, dehydration, demethanation and repolymerization reactions was achieved during HTC, showing no significant decrease in the mass yield [34]. A similar result was observed in hydrochar from waste eucalyptus bark by HTC, and mass yield ranged from 41.5 to 40.3% at 240°C for 4–6 h [23].

**Table 1.** Proximate analysis, HHV, and mass and energy yields of MNS and hydrochars.

	proximate analysis^a^ (wt%)							
samples	VM	FC	ash	HHV (MJ kg^−1^)	mass yield (%)	energy yield (%)	energy densification	BET surface area (m^2^ g^−1^)
MNS	77.68	19.81	2.51	18.78	—	—	—	0.2765
MNS220–60	69.62	27.62	2.76	22.75	63.73	77.20	1.21	3.9012
MNS220–120	66.81	29.91	3.28	23.99	56.81	72.57	1.28	5.3241
MNS220–180	63.56	32.47	3.97	24.68	52.22	68.63	1.31	11.6548
MNS180–120	71.58	25.44	2.98	21.67	72.42	83.56	1.15	2.6227
MNS260–120	54.98	40.81	4.21	26.02	46.59	64.55	1.39	12.4125

^a^VM, volatile matter; FC, fixed carbon; HHV, higher heating value.

Energy yield and densification are used to evaluate hydrochar produced from biomass [19,23]. As seen in table 1, energy densification increased with temperature from the minimum of 1.15 for MNS180–120 to the maximum of 1.39 for MSN260–120, denoting an increase of approximately 20.87%. However, a slight change in energy densification was found for hydrochar at 220°C for 60–180 min, and its increase was only 8.26%. This finding is due to the effect of HTC temperature during HTC exceeding that of residence time, and the thermal stability of MNS was destroyed at a slow rate at 220°C. The loss of oxygen and hydrogen content with the increase in temperature and residence time can promote the energy densification of hydrochar [29]. The results indicated that HTC can be treated as an applicable approach to upgrade the HHV of biomass. Energy yield decreased from 83.56 to 64.55% in the temperature range of 180–260°C. However, a slight difference in energy yield was observed for hydrochar produced at 220°C with an increase in residence time from 60 to 180 min. Cai *et al*. [35] previously reported similar results.

The surface area of the MNS and their hydrochar at different conditions is shown in table 1. The results of BET analysis showed that the BET surface area is improved and increased compared to that of the MNS. The BET surface is 12.4125 m^2^ g^−1^ at 260°C for 120 min. The increase in BET surface can be due to the reaction temperature and time, which caused the fibrous structure degradation, and produced the many pores in hydrochar.

### 3.2. Physico-chemical properties

Proximate analysis (table 1) showed that the volatile matter (VM) content of hydrochar decreased with the increase in HTC temperature because of dehydration and decarboxylation reactions [36], whereas FC content increased gradually. The VM and FC content of MNS were 77.68% and 19.81%, respectively. After HTC at 260°C for 180 min (MNS260–120), the VM content decreased to 54.98%, and the FC content increased to 40.81%. The decrease in VM between 220°C and 260°C was significantly higher than that between 180 and 220°C because of the decomposition of cellulose at 220°C [29]. The ash content of hydrochars increased gradually with the increase in the HTC temperature, indicating that the inorganics in ash were more stable than organic compounds during HTC. However, the results in the literature [37] show that inorganic materials can be efficiently removed from biomass after HTC because inorganics are partially dissolved in the liquid phase. The results of this study show that the effect of this phenomenon is extremely small. Table 1 shows that the residence time exhibited a similar trend in proximate analysis in contrast with HTC temperature.

HHV is one of the most important factors in the fuel properties of hydrochar. Table 1 shows that HHV increased from 18.78 MJ kg^−1^ for MNS to 21.67 MJ kg^−1^ for hydrochar at 180°C for 120 min, and further to 26.02 MJ kg^−1^ for hydrochar at 260°C for 120 min. The HHVs of hydrochars (21.67–26.02 MJ kg^−1^) all exceed that of lignite (20.89 MJ kg^−1^) [38], whereas the HHV of hydrochar at 260°C for 120 min was slightly higher than those of subbituminous coal (24.30 MJ kg^−1^) [39] and bituminous coal (25.84 MJ kg^−1^) [40]. The increase in HHV was due to the destruction of low-energy chemical bonds, and production of high-energy chemical bonds [22]. The results indicated that hydrochar from MNS can be used as a solid fuel.

Table 2 shows the ultimate analysis of hydrochar from different conditions. The C, H, O, N and S contents of raw MNS were 49.15%, 5.51%, 42.12%, 0.59% and 0.12%, respectively. The C content of hydrochar increased with the HTC temperature and reached 64.90% at 260°C for 120 min with the elevation of 32.0%. Meanwhile, the O content decreased, indicating that the fuel properties of MNS improved after HTC. C and O contents, respectively, increased and decreased gradually with the increase in residence time. This finding is attributed to decarboxylation, dehydration and demethanation reactions of biomass leading to the decline of H and O contents with H_2_, CH_4_, CO and CO_2_ evolving out as gas products [32,41]. H, N and S contents had no significant change after HTC.

**Table 2.** Ultimate analysis of MNS and hydrochars.

	ultimate analysis (wt%)					atomic ratio^a^	
samples	C	H	O^b^	N	S	O/C	H/C
MNS	49.15	5.51	42.12	0.59	0.12	0.64	1.35
MNS220–60	53.63	5.47	37.20	0.76	0.18	0.52	1.22
MNS220–120	55.68	5.41	34.78	0.68	0.17	0.47	1.17
MNS220–180	57.90	5.23	32.17	0.63	0.10	0.42	1.08
MNS180–120	51.32	5.44	39.47	0.69	0.10	0.58	1.27
MNS260–120	64.90	4.91	24.85	0.98	0.15	0.29	0.90

^a^O/C and H/C were given in atomic ratio.

^b^Oxygen content was obtained by difference.

**Figure 1.**
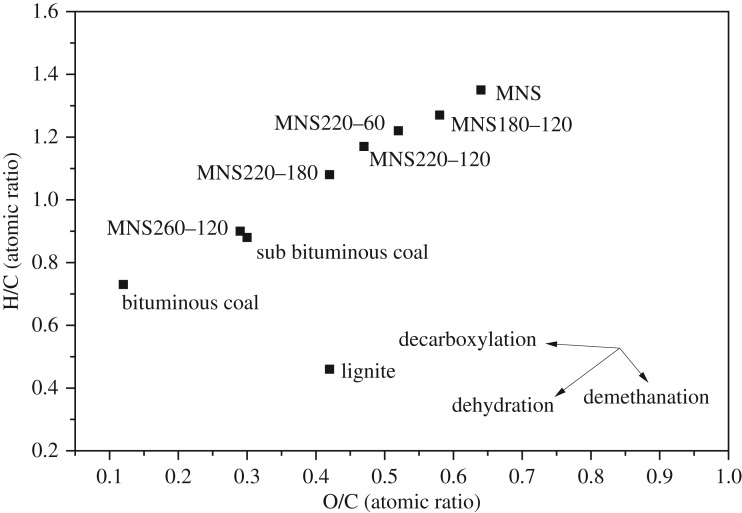
Van Krevelen diagram for MNS and hydrochars at different conditions. Three typical fuel coals including lignite, subbituminous coal and bituminous coal are shown for comparison.

The H/C and O/C atomic ratios of MNS and hydrochars were indicated in the Van Krevelen diagram (figure 1), which showed that the evolution of the H/C and O/C atomic ratios from MNS to hydrochars followed the paths of the decarboxylation and dehydration reactions. The H/C and O/C atomic ratios of lignite, subbituminous coals and bituminous coals were also plotted in the Van Krevelen diagram for comparison (figure 1). Figure 1 shows that the H/C and O/C atomic ratios of hydrochar were in the range of 0.90–1.27 and 0.29–0.58, respectively, which were lower than those of raw MNS. With the increase in HTC temperature and residence time, the H/C and O/C atomic ratios of hydrochars approached those of subbituminous, and bituminous coals.

### 3.3. Fourier transform infrared spectroscopy analysis

**Figure 2.**
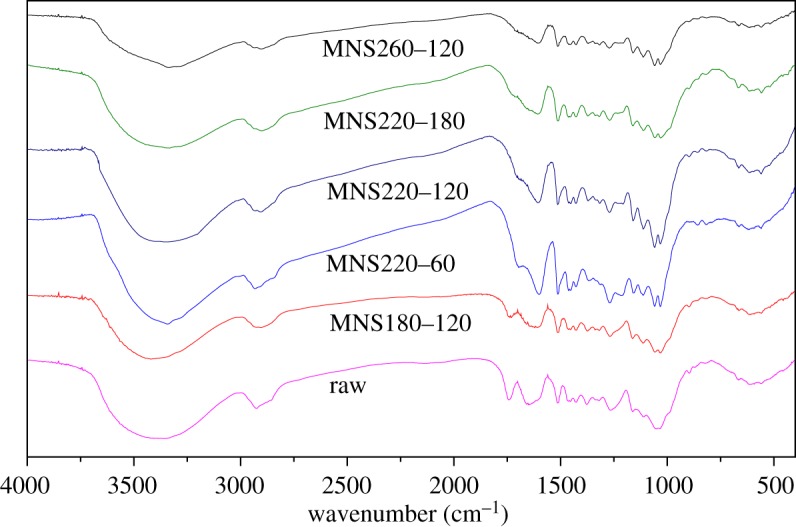
FTIR spectra of MNS and hydrochars.

The FTIR spectra of raw MNS and hydrochars produced under different conditions are shown in figure 2. The spectra can be used to evaluate the evolution of functional groups in hydrochars from different HTC and residence times [42]. The FTIR spectra of hydrochars were different from that of raw MNS, indicating that chemical reactions occurred during HTC. The band at 3385 cm^−1^ was attributed to the O–H stretching vibration in hydroxyl and carboxyl groups. With the increase in HTC temperature, this peak became less intense than that of raw MNS, mainly because of the degree of deoxygenation and dehydration during HTC [15]. Moreover, the decrease in hydroxyl and carboxyl contents can increase the hydrophobicity of hydrochar [43,44]. Bands at 2910 and 2830 cm^−1^ were associated with C–H stretching vibration in aliphatic and aromatic structures [23]. Slight change in the C–H stretching vibration of hydrochars was found, except for hydrochar at 260°C for 120 min. The results indicated that the demethanation reaction occurred at 260°C for 120 min during HTC. The band at 1710 cm^−1^ was associated with the C=O stretching vibration in ketone, amide and carboxyl groups [23]. Absorbance intensity weakened with the increase in HTC temperature because of decarboxylation and dehydration reactions during HTC. Bands at 1605, 1510 and 1465 cm^−1^ were attributed to the C=C stretching vibration in the benzene ring skeleton [32]. The band at 1025 cm^−1^ was associated with the C–O stretching vibration [23]. The intensity of C=C and C–O stretching vibrations was weakened with the increase in HTC temperature, indicating that lignin was partially decomposed. Figure 2 shows that the effect of different residence times on functional group in hydrochars was less significant, indicating that the dominant HTC reaction process was nearly completed during the first 60 min.

### 3.4. Scanning electron microscopy analysis

**Figure 3.**
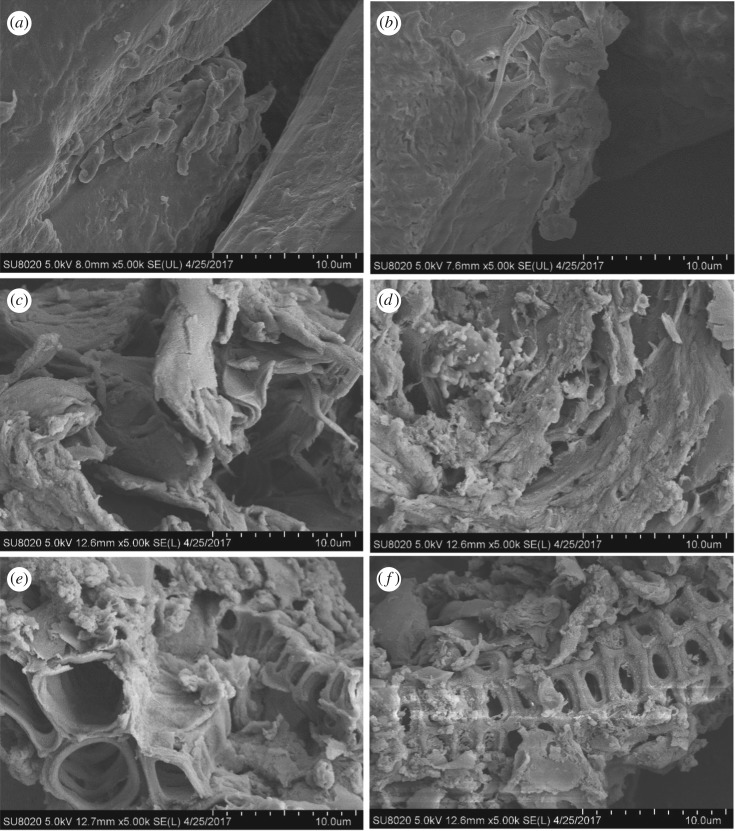
SEM image of MNS and hydrochars: (*a*) raw MNS, (*b*) MNS180–120, (*c*) MNS220–60, (*d*) MNS220–120, (*e*) MNS220–180 and (*f*) MNS260–120.

Figure 3 shows the physical structure of raw MNS and hydrochar. The surface morphology and physical structure of hydrochars and raw MNS are different at different HTC conditions. Raw MNS showed a smooth surface structure (figure 3*a*). After HTC treatment, the surface morphology and physical structure of hydrochars become rougher with the increase in HTC temperature and residence time; however, different findings were observed in hydrochar produced at 180°C for 120 min (figure 3*b*). This phenomenon is attributed to the decomposition of hemicellulose, cellulose and lignin [29,45] above 220°C. At 180°C for 120 min, biomass degradation occurred at the surface of the biomass because of mild HTC. With the increase in HTC temperature, small pores and fragments were gradually formed on the hydrochars' surface because of the VM release process. Moreover, the regular pore structures were found at 220°C for 180 min and 260°C for 120 min because elevated temperature and increased residence time enhanced the decomposition of the cellulose and hemicellulose. On the other hand, a small amount of lignin components was decomposed under these HTC conditions. These degraded components leave regular pore structures on the samples.

### 3.5. Combustion behaviour

**Figure 4.**
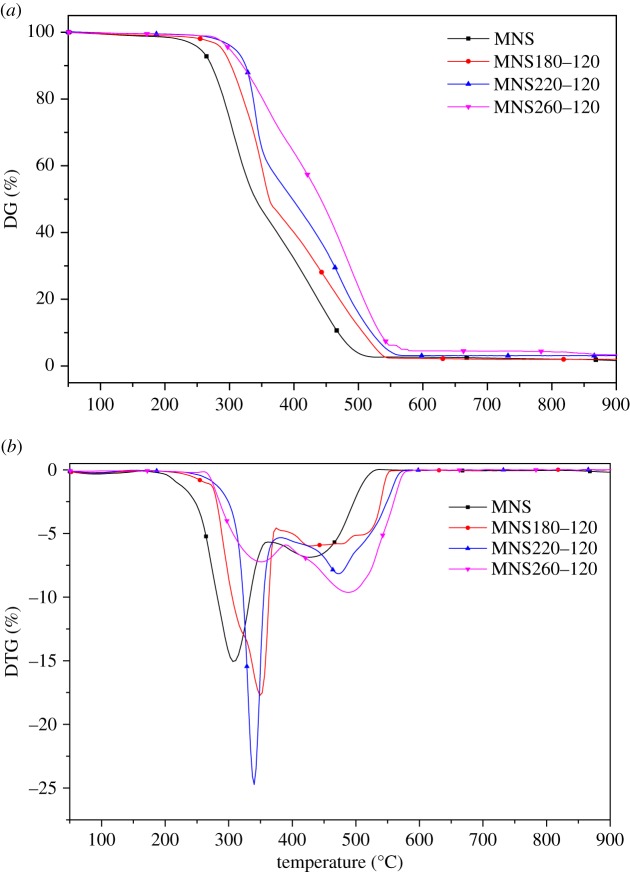
TG and differential thermogravimetric curves of MNS and hydrochars.

Given that temperature is the main factor affecting HTC of MNS, the combustion behaviour of hydrochars was only analysed on the basis of hydrochars produced at 180, 220 and 260°C for 120 min. Figure 4 shows the TG and derivative TG (DTG) curves of MNS and hydrochars. The combustion behaviours of MNS resulted insubstantial changes in hydrochars produced via HTC. The main combustion processes of MNS and hydrochars were divided into two stages to evaluate the combustion characteristics further. Compared with raw MNS, the weight loss rate of MNS180–120 and MNS220–120 increased at the first peak, whereas that of MNS260–120 decreased. At the second peak, the hydrochar produced at 260°C exhibited the largest weight loss rate because the hydrochars produced at 260°C contained lesser VM than those produced at 180 and 220°C. The first peak occurred because of the combustion of VM, and the second peak resulted from the combustion of FC [15]. The weight loss rate of MNS was lower than those of MNS180–120 and MNS220–120. This phenomenon was due to the low ignition temperature of MNS than that of biochars, resulting in a wide range of combustion temperatures. The results of the study are in agreement with Ma *et al.* [46].

## 4. Conclusion

HTC of MNS increased the HHV of hydrochar, indicating that MNS can be used as a high-quality energy source via HTC. With the increase in HTC temperature and residence time, hydrochars showed superior physico-chemical properties compared with raw MNS. The HHV is 26.02 MJ kg^−1^ at 260°C for 120 min, but mass and energy yield were only 46.59% and 64.55%, respectively. The FTIR analysis confirmed that dehydration and decarboxylation occurred in the HTC process. The surface morphology of hydrochars was rougher than that of MNS, and the regular pore structures were found at 220°C for 180 min and 260°C for 120 min. The TG and DTG results showed that the combustion behaviour of MNS and hydrochars are different because of differences in VM and FC contents.

## Data accessibility

This article does not contain any additional data.
